# Effects of 12-week gait retraining on plantar flexion torque, architecture, and behavior of the medial gastrocnemius *in vivo*

**DOI:** 10.3389/fbioe.2024.1352334

**Published:** 2024-03-20

**Authors:** Chuyi Zhang, Liqin Deng, Xini Zhang, Kaicheng Wu, Jianglong Zhan, Weijie Fu, Jing Jin

**Affiliations:** ^1^ School of Exercise and Health, Shanghai University of Sport, Shanghai, China; ^2^ Faculty of Sports Science, Ningbo University, Ningbo, China; ^3^ Key Laboratory of Exercise and Health Sciences of Ministry of Education, Shanghai University of Sport, Shanghai, China; ^4^ School of Psychology, Shanghai University of Sport, Shanghai, China

**Keywords:** gait retraining, medial gastrocnemius, plantar flexion torque, muscle architecture, ultrasound

## Abstract

**Objective:**

This study aims to explore the effects of 12-week gait retraining (GR) on plantar flexion torque, architecture, and behavior of the medial gastrocnemius (MG) during maximal voluntary isometric contraction (MVIC).

**Methods:**

Thirty healthy male rearfoot strikers were randomly assigned to the GR group (*n* = 15) and the control (CON) group (*n* = 15). The GR group was instructed to wear minimalist shoes and run with a forefoot strike pattern for the 12-week GR (3 times per week), whereas the CON group wore their own running shoes and ran with their original foot strike pattern. Participants were required to share screenshots of running tracks each time to ensure training supervision. The architecture and behavior of MG, as well as ankle torque data, were collected before and after the intervention. The architecture of MG, including fascicle length (FL), pennation angle, and muscle thickness, was obtained by measuring muscle morphology at rest using an ultrasound device. Ankle torque data during plantar flexion MVIC were obtained using a dynamometer, from which peak torque and early rate of torque development (RTD_50_) were calculated. The fascicle behavior of MG was simultaneously captured using an ultrasound device to calculate fascicle shortening, fascicle rotation, and maximal fascicle shortening velocity (V_max_).

**Results:**

After 12-week GR, 1) the RTD_50_ increased significantly in the GR group (*p* = 0.038), 2) normalized FL increased significantly in the GR group (*p* = 0.003), and 3) V_max_ increased significantly in the GR group (*p* = 0.018).

**Conclusion:**

Compared to running training, GR significantly enhanced the rapid strength development capacity and contraction velocity of the MG. This indicates the potential of GR as a strategy to improve muscle function and mechanical efficiency, particularly in enhancing the ability of MG to generate and transmit force as well as the rapid contraction capability. Further research is necessary to explore the effects of GR on MG behavior during running *in vivo*.

## 1 Introduction

Running has become an important sport and promotes the development of global fitness activities (Hulteen et al., 2017). The medial gastrocnemius (MG) is a biarticular muscle spanning the knee and ankle joint and essential for transmitting force and power during the absorption and push-off phases of running (Lai et al., 2018; Moran et al., 2023). The MG and the lower tendon complete the storage and release of elastic strain energy, thereby improving the mechanical efficiency of running (Yong et al., 2020). Muscle architecture, including fascicle length (FL), pennation angle (PA), and muscle thickness (MT), is a major determinant of muscle functional characteristics (Murach et al., 2015). Specifically, FL (the number of sarcomere arranged in series) and PA determine muscle length and the length range of force generation, which in turn influence the shortening velocity of muscles and capacity to generate force (Kruse et al., 2021; May et al., 2021). PA and MT are considerably related to maximum muscle strength (Secomb et al., 2015; May et al., 2021). During running, the muscle bundles of the MG actively shorten and rotate to attenuate impact force, provide support, and cause propulsion (Hamner et al., 2010; Ahn et al., 2014; Eng et al., 2018). The functions of the muscle, including cushioning, muscle force generation, and power output, and metabolic costs are influenced to some extent by fascicle shortening and fascicle rotation (Ishikawa et al., 2007; Deng et al., 2023). Consequently, the architecture and behavior of MG play a crucial role in running by influencing muscle function.

With the development of cushioned running shoes, researchers found that 95.1% of recreational shod runners run with a rearfoot strike pattern (RFS) (de Almeida et al., 2015). Considerable differences in the biomechanics of lower limb joints have been found among different foot strike patterns over the last 10 years (Almeida et al., 2015; Yong et al., 2020; Xu et al., 2021; Ye et al., 2024). Compared with runners with a RFS, those with a forefoot strike pattern (FFS) exhibit greater knee flexion and ankle plantar flexion during initial contact (Almeida et al., 2015) and greater plantar flexion torque during the early stance phase (Kulmala et al., 2013; Gonzales et al., 2019). The activation of the MG greatly increased during FFS compared with that in RFS (Lin et al., 2021), indicating increased mechanical loading on the MG during running with FFS. Moreover, the excessive eccentric loading during ground contact in FFS may affect the stretch-shortening cycle of the triceps surae muscle–tendon unit, which in turn affects the ability to resist impact force during landing and return elastic energy (Jewell et al., 2017). Significant differences in MG morphology have been found among different habitual foot strike patterns (Li et al., 2023). Specifically, FFS runners have longer FL and smaller PA; these changes increase maximal shortening velocity and efficiency of force transmission. These biomechanical differences collectively reveal that the MG is influenced by the foot strike pattern during running, including muscle activity, mechanical properties, and elastic energy utilization, thereby affecting running performance and mechanical efficiency.

Gait retraining (GR) has been applied to the transition from RFS to FFS in running (Deng et al., 2020; Wang et al., 2020; Yang et al., 2020). By conducting GR on RFS runners for 12 weeks, GR effectively reduced the vertical loading rate and prevented vertical peak impact forces, consequently reducing the risk of injury related to impact force (Yang et al., 2020). A study on the immediate transition of RFS runners to running with FFS revealed that compared with RFS, FFS exhibited markedly decreased knee extension torque and increased plantar flexion torque (Kuhman et al., 2016). Similar results were obtained in studies focusing on GR (Deng et al., 2020; Wang et al., 2020). In our previous study, we revealed that after a 12-week GR intervention, the increased plantar flexion torque resulted in the loading of the triceps surae muscle–tendon unit, effectively enhancing its mechanical properties (Zhang et al., 2021). In summary, we speculated that the elevated mechanical loading from gradual GR can be conducive to adaptive changes in the architecture of MG, so as to improve mechanical efficiency and physical performance.

Therefore, this study aimed to investigate the effects of a 12-week GR program on plantar flexion torque, architecture, and behavior of MG during the maximal voluntary isometric contraction (MVIC) *in vivo* by using ultrasound and an isokinetic dynamometer. We hypothesized that after GR, the majority of rearfoot strikers would transition to non-RFS while running. Besides, the GR group would exhibit the following changes: 1) significant increase in the peak torque of plantar flexion and early rate of torque development during MVIC; 2) significant increase in FL and MT of MG at rest, along with a significant decrease in PA of MG; 3) significant increase in fascicle shortening, fascicle rotation, and maximal fascicle shortening velocity of MG during the MVIC.

## 2 Materials and methods

### 2.1 Participants

Thirty male, healthy rearfoot strike runners (age: 33.3 ± 8.8 years; height: 173.7 ± 5.8 cm; body mass: 70.0 ± 8.4 kg; weekly running distance: 40.4 ± 18.9 km) were selected, and they were randomly allocated into the GR group (*n* = 15) and control (CON) group (*n* = 15). A post-power analysis (G*Power version 3.1, Kiel University, Kiel, Germany) was conducted, and *n* = 24 (total sample size) would provide a power of 0.99 for the effect size of normalized FL in this study (effect size f = 0.445). The inclusion criteria were as follows: 1) male runners who are used to running with RFS in cushioned shoes and have never tried minimalist shoes, 2) do not have a history of lower extremity injuries within the previous 6 months or neuromuscular diseases, and 3) have run at least 20 km per week in the past 3 months and intend to maintain training intensity in the next 12 weeks. Prior to the study, all participants signed an informed consent form approved by the Ethics Committee of Shanghai University of Sport (No. 102772021RT085).

### 2.2 Instrumentation

**FIGURE 1:**
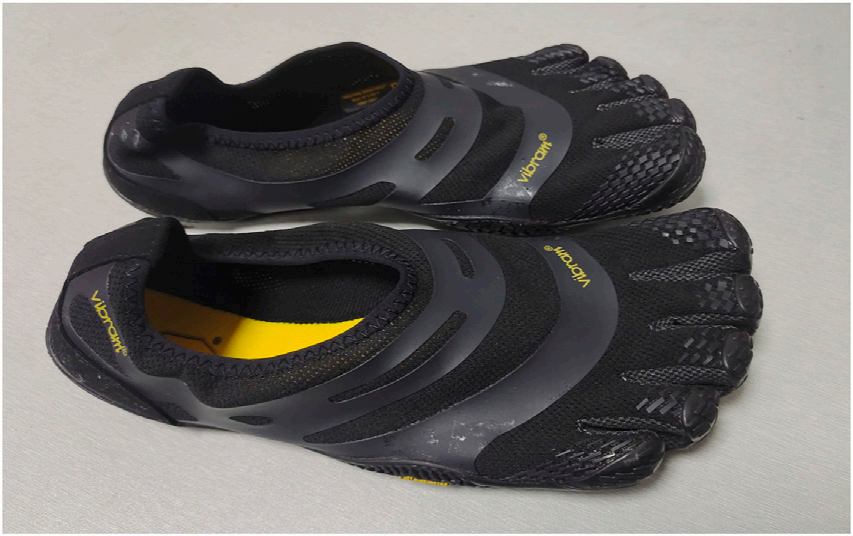
Vibram five-finger minimalist shoes.

An ultrasonography system (22 Hz, uSmart 3,300, Terason, United States) with a 12L5A linear array probe (12 MHz maximum frequency) was used to capture MG ultrasound images. A dynamometer (256 Hz, Con-Trex MJ, Physiomed, Germany) was used to measure the plantar flexion torque during MVIC. During the intervention process, participants in the GR group wore Vibram five-finger minimalist shoes (3 mm outsole, 0 mm heel-to-toe drop, no midsole, average mass of 139 g, Figure 1).

### 2.3 Experimental procedure

**FIGURE 2:**
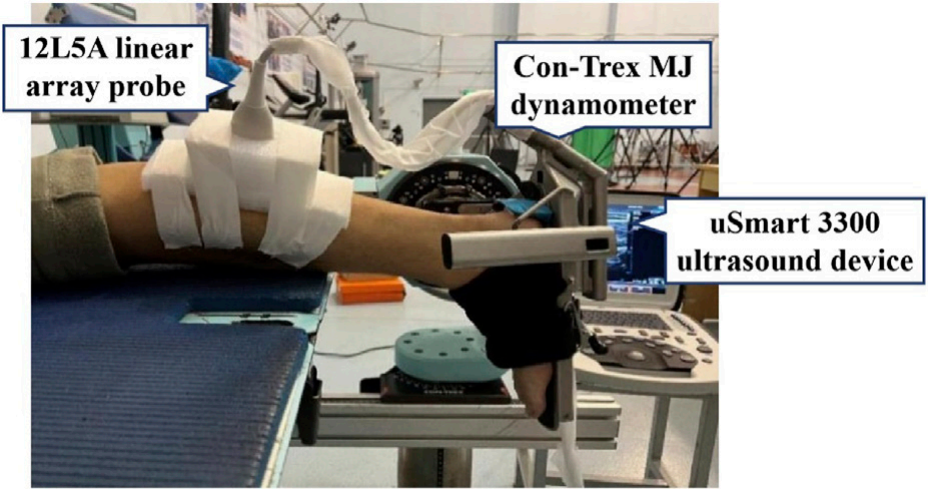
The measurement location during MVIC.

To warm up, each participant ran for 5 minutes on a treadmill at a self-selected speed according to their habitual strike pattern (Deng et al., 2023). Simultaneously, the researcher utilized a mobile phone to record running videos of the participants during warm-up, aiming to initially verify the strike pattern they had self-reported. At the beginning of the test, the participants lay prone on a treatment bed, with the ankle in a neutral position (forming a 90° angle between the lower leg and foot) and the knee and hip fully extended. The experimenter applied ultrasound gel to the head of the probe head and placed the probe at 30% of the distance between the popliteal crease and the lateral malleolus to obtain morphological images of the MG at rest (Geremia et al., 2018). Three clear morphological images of the MG were recorded for data analysis. Participants were then required to be seated on the treatment bed, ensuring that the ankle was in a neutral position and the knee and hip were flexed at 90°. The shank length was defined as the distance from the medial tibial condyle to the medial malleolus of the ankle and measured with a measuring tape (Deng et al., 2021). During the MVIC test, the participants were prone on the dynamometer with the hip and knee extended, and the ankle was fixed in a neutral position. Subsequently, the participants contracted as hard as possible from a relaxed state to the maximum isometric contraction state of plantar flexion within 5 seconds to obtain the torque of the ankle (Deng et al., 2020). This action was repeated three times. Meanwhile, an ultrasound probe was secured to the MG belly of the participants using an elastic bandage to record a video of the *in vivo* behavior changes of the MG (Figure 2). After all the tests, the participants were trained according to the results of different groups, and the above tests were repeated after 12 weeks.

### 2.4 Intervention

Participants in the GR group were instructed to wear five-finger minimalist shoes for the 12-week GR. During GR, participants were asked to adopt an FFS at a self-selected and moderate speed. The metatarsal heads of the forefoot were required to make initial ground contact, followed by the remaining parts of the foot, while the foot landed below the hip (Wang et al., 2020). The participants were required to maintain their weekly running volume to be consistent with their original running volume. The running distance for GR only replaced a certain portion of the total running distance, whereas the remaining portion was trained according to the original running habits. The running distance of FFS running with five-finger minimalist shoes increased progressively (Table 1). Specifically, the participants performed 10% of the weekly running volume with five-finger minimalist shoes in weeks 1 and 2. The running distance of GR increased by 10% every week from weeks 3–10 until it was completed at 100% during weeks 11 and 12 (Joseph et al., 2017). The 12-week GR program involved participants attending three sessions per week. All participants underwent the intervention under the supervision of the experimenters, and their foot strike pattern was corrected and coached by professional long-distance running athletes who were accustomed to running with an FFS. For 12 weeks, the participants were required to perform foot core exercises targeting foot function and muscle strength. These exercises were designed to prevent the musculoskeletal system of the foot and ankle complex from adapting to the mechanical loads associated with long-term GR (Wang et al., 2020). The foot core exercises in this study consisted of bilateral heel raises, unilateral heel raise, towel curls, short foot exercise, and toe spread and squeeze (20 min per time). Every 2 weeks, exercise intensity was varied by increasing the difficulty and quantity of motions (Table 2). The detailed intervention program and monitoring modality were based on our previous study (Zhang et al., 2024).

**TABLE 1:** 12-week gait retraining (GR) program.

Week	1	2	3	4	5	6	7	8	9	10	11	12
Running distance (% weekly running volume)	10	10	20	30	40	50	60	70	80	90	100	100
Times per week	3	3	3	3	3	3	3	3	3	3	3	3

**TABLE 2:** 12-week foot core exercise program.

Week	1–2	3–4	5–6	7–8	9–10	11–12
Bilateral heel raises (level surface)	3 × 20	3 × 20	3 × 20	3 × 20	3 × 20	3 × 20
Bilateral heel raises (on step)	N/A	3 × 20	3 × 20	3 × 20	3 × 20	3 × 20
Unilateral heel raise (level surface)	N/A	N/A	3 × 10	3 × 15	3 × 20	3 × 20
Towel curls	3 × 20	3 × 20	3 × 30 (0.25 kg)	3 × 30 (0.25 kg)	3 × 30 (0.5 kg)	3 × 30 (0.5 kg)
Short foot exercise	3 × 20	3 × 20	3 × 20	3 × 30	3 × 30	3 × 30
Toe spread and squeeze	3 × 20	3 × 20	3 × 20	3 × 30	3 × 30	3 × 30

In the CON group, the participants were instructed to continue wearing their habitual cushioned running shoes and continue the running training by using their original foot strike pattern while maintaining the same exercise intensity as before the experiment.

### 2.5 Data analysis

The plantar flexion torque was obtained directly by using a dynamometer. The peak torque of the plantar flexion (PT) was the maximum value of torque generated by the participant during MVIC, which was then normalized by body weight. The rate of torque development in the early 50 ms (RTD_50_) was calculated as the average slope of the time–torque curve 0–50 ms from the onset of the contraction (Andersen et al., 2010). The onset of plantar flexion was defined as the instant at which the torque exceeded the baseline by 3% of the PT (Trajković et al., 2021).

The data of the MG architecture (FL, PA, and MT) at rest were processed by Image J software (NIH, Bethesda, MD, United States). The FL was defined as the length of the fascicular pathway between the superficial and deep fascias, and it was determined by calculating the average length of three fascicles in an ultrasound image (Geremia et al., 2019). The normalized FL was obtained by dividing the FL by the shank length (Li et al., 2023). The PA was defined as the angle formed between the muscle fascicle and deep fascia, the mean value of which was calculated according to the three ultrasound images of each participant (Geremia et al., 2019). The MT was defined as the vertical distance from the deep aponeurosis to the superficial aponeurosis, and it was computed by calculating the average length of the five perpendicular parallel lines drawn between the two aponeuroses (Geremia et al., 2019).

Ultratrack software (version 4.1) was used in determining the intersection of fascicles on the superficial and deep fascias on the ultrasound video of MG during MVIC with semi-automated tracking and manual correction (Swinnen et al., 2022). The fascicle shortening (∆L) was computed as the change in the FL of the MG from at rest to MVIC. The fascicle rotation (∆θ) was calculated as the absolute change in PA of the MG from at rest to MVIC. The maximal fascicle shortening velocity (V_max_) was computed as the maximal slope of the FL–time curve during MVIC (Hauraix et al., 2017).

The foot strike angle in this study was obtained by calculating the relative angle between the foot and the ground at the time of initial ground contact, which is the difference between the angle of the foot at touchdown and the angle of the foot when standing. Detailed information on biomechanical testing during running can be found in our previous study (Zhang et al., 2024).

### 2.6 Statistics

Data were presented as mean ± standard deviation. The normality of the data distribution was analyzed using the Shapiro–Wilk test. For parameters that did not conform to the normal distribution, a logarithmic transformation was applied to achieve normality. A two-way repeated measures ANOVA (group × time) was used in examining the effects of the 12-week GR on the plantar flexion torque, architecture, and behavior of MG (version 23.0, SPSS Inc., Chicago, IL, United States). For parameters with an interaction effect between time and group, a simple effects analysis was performed as a *post hoc* test. The significance level (*α*) was set at 0.05.

## 3 Results

### 3.1 Dropout rate

Twenty-four participants completed the 12-week intervention, and the results were included in the statistical analysis (Table 3). Six participants (three in the GR group and three in the CON group) were excluded, and the dropout rate was 20%. Specifically, during the intervention, one participant in the CON group was excluded for trying to run while wearing five-finger minimalist shoes. One participant in the GR group was excluded because of personal reasons and his inability to participate in the test after training. Four participants (two in the GR group and two in the CON group) were excluded because of the absence of intervention for more than 2 weeks. In the GR group (comprising 12 rearfoot strike runners), 10 participants transitioned to non-RFS, and thus the transition rate was 83.3%. No significant differences in age, height, weight, or weekly running volume were found between the two groups. Given that the normalized PT, RTD_50_, and V_max_ did not conform to the normal distribution, the data were logarithmically transformed before statistical analysis.

**TABLE 3:** Basic information of included participants (*n* = 24).

Group	Age (years)	Height (cm)	Body Mass (kg)	Weekly running volume (km)
GR (*n* = 12)	34.75 ± 8.71	174.00 ± 6.94	71.64 ± 6.44	40.42 ± 14.84
CON (*n* = 12)	32.58 ± 10.04	172.17 ± 5.49	68.48 ± 9.94	42.58 ± 25.64
*p*-value	0.578	0.481	0.365	0.887

Notes: GR, gait retraining; CON, control.

### 3.2 Ankle torque during MVIC

**FIGURE 3:**
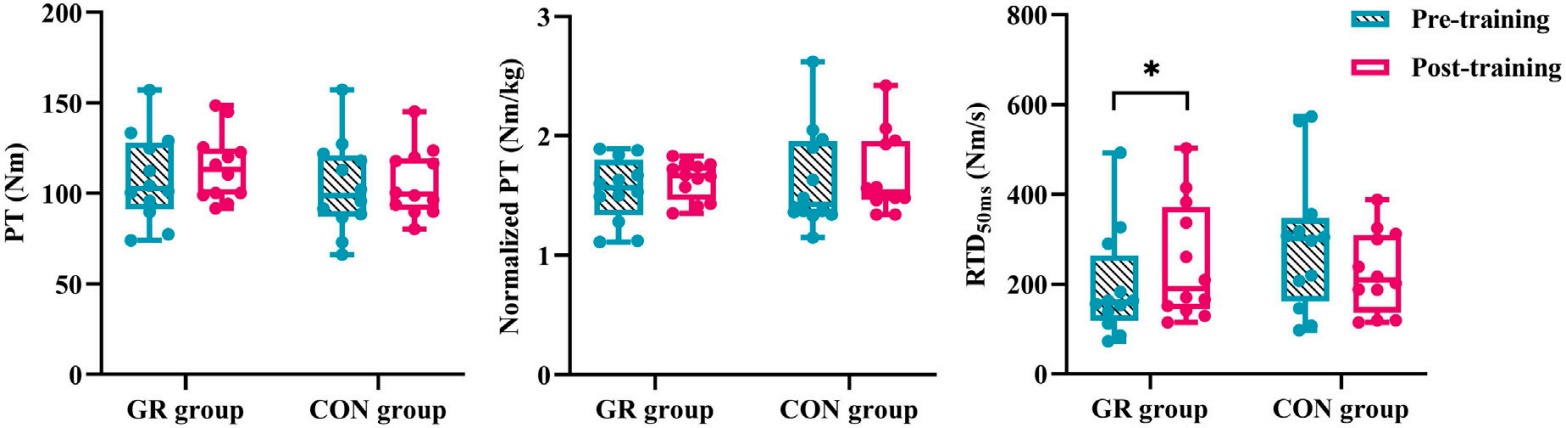
Effects of 12-week GR on peak torque of plantar flexion and early rate of torque development during MVIC. GR: gait retraining; CON: control; RTD_50_: rate of torque development during the 0–50 ms period of MVIC. * indicates a significant difference in pre- and post-training, *p* < 0.05.

A significant interaction effect between time and group was observed in the RTD_50_ (*p* = 0.014, Figure 3). The *post hoc* test showed that the RTD_50_ of the GR group after training significantly increased compared with that before training (*p* = 0.038). Meanwhile, no significant main effect or interaction effect was observed for PT or normalized PT (*p* > 0.05, Table 4).

**TABLE 4:** Effects of 12-week gait retraining (GR) on the plantar flexion torque during MVIC and architecture and behavior of MG.

Parameter	GR group		CON group		*p*-value ( $\eta^{2}$ )		
	PRE	POST	PRE	POST	Main effect for time	Main effect for group	Interaction effect
PT (N/m)	108.33 ± 24.30	114.45 ± 18.84	103.44 ± 25.44	106.08 ± 18.60	0.102 (*0.117*)	0.450 (*0.026*)	0.503 (*0.021*)
Normalized PT (Nm/kg)	1.55 ± 0.27	1.63 ± 0.16	1.63 ± 0.43	1.68 ± 0.34	0.069 (*0.143*)	0.698 (*0.007*)	0.661 (*0.009*)
RTD_50_ (Nm/s)	195.11 ± 119.69	248.61 ± 129.99*	291.75 ± 154.91	226.24 ± 89.31	0.660 (*0.009*)	0.361 (*0.038*)	**0.014 (*0.247*)**
FL (cm)	6.39 ± 0.65	7.00 ± 0.78	6.45 ± 0.93	6.54 ± 0.88	**0.012 (*0.257*)**	0.524 (*0.019*)	0.058 (*0.154*)
Normalized FL	0.19 ± 0.03	0.21 ± 0.03*	0.20 ± 0.03	0.20 ± 0.03	**0.015 (*0.242*)**	0.948 (*0.000*)	**0.049 (*0.165*)**
PA (°)	18.76 ± 1.46	18.40 ± 1.70	18.48 ± 3.09	18.13 ± 2.60	0.368 (*0.037*)	0.752 (*0.005*)	0.982 (*0.000*)
MT (cm)	1.79 ± 0.27	1.81 ± 0.27	1.80 ± 0.28	1.74 ± 0.23	0.679 (*0.008*)	0.804 (*0.003*)	0.334 (*0.042*)
∆L (cm)	2.08 ± 0.65	2.58 ± 0.57	1.94 ± 0.60	2.28 ± 0.50	**0.001 (*0.415*)**	0.307 (*0.047*)	0.456 (*0.026*)
∆θ (°)	12.64 ± 5.33	16.88 ± 5.72	13.12 ± 6.69	14.26 ± 5.56	**0.003 (*0.331*)**	0.638 (*0.010*)	0.071 (*0.140*)
V_max_ (cm/s)	6.15 ± 3.05	9.27 ± 5.22*	9.41 ± 4.94	7.48 ± 3.89	0.457 (*0.025*)	0.595 (*0.013*)	**0.009 (*0.269*)**

Notes: PT, peak torque of plantar flexion; RTD_50_, rate of torque development during the 0–50 ms period of MVIC; FL, fascicle length at rest; PA, pennation angle at rest; cMT, muscle thickness at rest; V_max_, maximal fascicle shortening velocity during MVIC; ∆L, fascicle shortening during MVIC; ∆θ, fascicle rotation during MVIC.

* *p* < 0.05: significant difference in pre- and post-training. The bold values indicate that the parameter has a significant main effect of time or an interaction between group and time.

### 3.3 Architecture and behavior of MG

**FIGURE 4:**
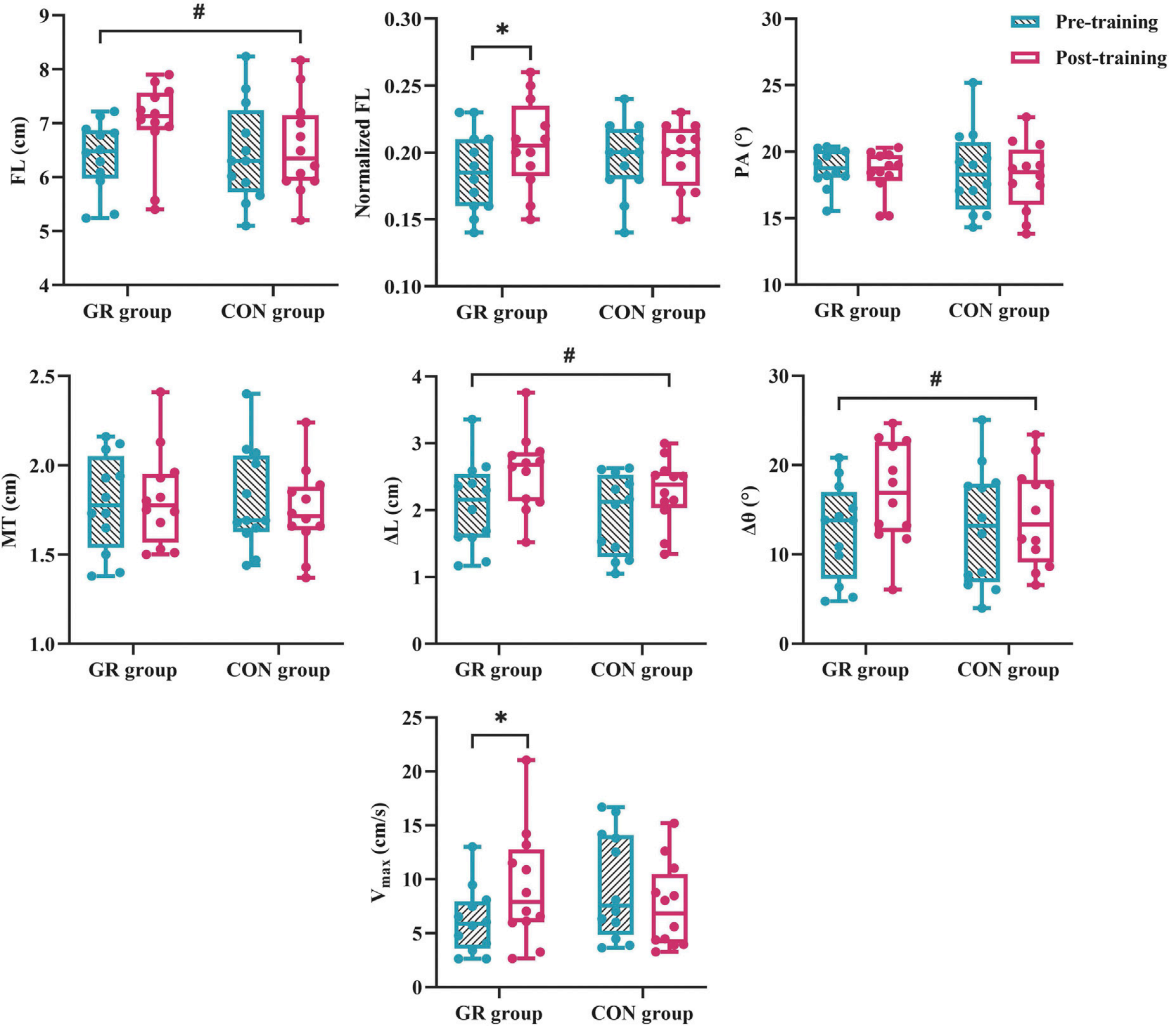
Effects of 12-week GR on the architecture and behavior of MG *in vivo*. GR: gait retraining; CON: control; FL: fascicle length at rest; PA: pennation angle at rest; MT: muscle thickness at rest; V_max_: maximal fascicle shortening velocity during MVIC; ∆L: fascicle shortening during MVIC; ∆θ: fascicle rotation during MVIC. # indicates a significant main effect of time. * indicates a significant difference in pre- and post-training, *p* < 0.05.

For MG architecture, an interaction effect between time and group (*p* = 0.049) and a significant main effect of time (*p* = 0.015) were observed in the normalized FL. The *post hoc* test showed that the normalized FL of the GR group increased significantly after training compared with that before training (*p* = 0.003). A significant main effect of time was observed in the FL (*p* = 0.012). Specifically, FL increased by 9.8% (GR) and 2.2% (CON) after training (Figure 4). No significant main effect or interaction effect was observed in the PA or MT (*p* > 0.05, Table 4).

For MG behavior, an interaction effect was found between time and group for the V_max_ of the MG during plantar flexion MVIC (*p* = 0.009). The *post hoc* test showed that the V_max_ of the GR group increased significantly after training compared with that before training (*p* = 0.018). A significant main effect of time was observed in ∆L (*p* = 0.001) and ∆θ (*p* = 0.003). Specifically, ∆L increased by 30.1% (GR) and 25.4% (CON), and ∆θ increased by 44.9% (GR) and 23.0% (CON) (Figure 4).

## 4 Discussion

This study examined the effects of a 12-week gradual GR on plantar flexion torque during isometric contraction, MG architecture, and behavior during MVIC *in vivo*. After 12 weeks, the GR group exhibited a significant increase in RTD_50_, normalized FL, and V_max_. FL, ∆L, and ∆θ increased in both groups. Inconsistent with our hypothesis, no significant changes in PT, PA, and MT were observed after GR.

The RTD_50_ of the plantar flexion during MVIC in the GR group markedly increased after training. This result was consistent with our previous findings. After 12 weeks of GR while wearing minimalist shoes, there was an increasing trend in peak RTD of plantar flexion (Deng et al., 2020). This result may be attributed to the short contact time during FFS running (Sun et al., 2018), which requires the plantar flexors to generate torque rapidly to resist the loading of the ankle joint. As a basic element of strength quality, RTD is typically manifested in the stretch-shortening cycle. RTD plays a crucial role during fast movements, which is closely related to maximal strength output and fascicle shortening velocity (Werkhausen et al., 2022; Dalton et al., 2023). The increased RTD observed after GR indicated an improved ability to achieve a high level of strength during the early phase of contraction. The ability of runners to rapidly develop strength affects contact time during long-distance running. A large RTD indicates capacity to generate the required strength in a short contact time, thereby influencing running performance (Lum et al., 2020). Therefore, gradual GR can be an effective mechanical stimulus that improves the rapid contraction ability of the MG during the early push-off phase. Although no significant change in PT was observed, we found a tendency for normalized PT to increase in both groups after training (*p* < 0.1). Previous studies demonstrated that PT is significantly greater in FFS than in RFS (Kulmala et al., 2013; Melcher et al., 2017; Gonzales et al., 2019). A large PT contributes to providing sufficient propulsion during running, accelerating the forward movement of the body (Gonzales et al., 2019). Given the 12-week duration of GR in our study, it might be necessary to further consolidate the effects of GR through long-term training, yet this requires further investigation.

The study found that the normalized FL increased significantly in the GR group after training, and the FL of the MG increased significantly in both groups after training. A prior study has also provided evidence that long-term running with FFS could significantly influence the architecture of MG, resulting in runners having a longer FL (Li et al., 2023). Compared with running with RFS, running with FFS requires more active involvement from the MG (Lin et al., 2021). This condition may increase the range of muscle stretching and contraction during running, resulting in adaptive changes in the muscle and an increase in FL. FL is a major factor in dictating muscle contraction velocity (Wickiewicz et al., 1984; Abe et al., 2000), and a long FL can result in shortening velocity and mechanical power greater than those in a short FL (Arampatzis et al., 2006). Theoretically, a longer FL is considered to have more serially linked sarcomeres (Cigoja et al., 2022), and the simultaneous contraction of these sarcomeres can result in a large overall change in FL. The result suggested that adaptive changes in the FL of MG after GR may play an important role in increasing the velocity of contraction. Longer fascicles also facilitate a slower shortening of sarcomeres within the muscle fibers, allowing the muscle to operate higher on its force-velocity curve and generate more force during contraction (Lin and Pandy, 2022). This indicated that the increased normalized FL after GR may benefit RTD, which was supported by the results of this study showing increased RTD_50_ after GR. Moreover, short fascicles are associated with an increased risk of microscopic muscle damage during repetitive eccentric actions in running (Timmins et al., 2016). From the perspective of muscle architecture, despite the increased demand on the triceps surae with the FFS, the change in FL suggests that GR can potentially reduce the risk of injury associated with high mechanical stimulation by lengthening fascicles. The absence of a significant difference in FL between the GR and CON groups might be attributed to the influence of variability in leg length.

The result showed a significant increase in V_max_ during MVIC in the GR group after 12 weeks. This increase can be attributed to increased FL, broadening the operating range of active muscle within the length–tension relationship and thereby resulting in an increased V_max_ of MG (Lieber and Friden, 2000). However, it is essential to emphasize that in this study, V_max_ measurement was conducted during MVIC and did not assess MG behavior during running. A previous study reported that forefoot strikers exhibited a slower fascicle contraction velocity of MG during ground contact compared to rearfoot strikers (Swinnen et al., 2019). Therefore, future research should prioritize investigating the behavioral changes of MG during actual running *in vivo*. The force–velocity relationship is a crucial factor influencing the performance of muscle contraction, and the maximum power output of muscles is constrained by this relationship (Daley et al., 2009; Fletcher and MacIntosh, 2017). For a given required muscle strength, the maximum power output increased with V_max_. In this study, V_max_ significantly increased after GR, whereas PT had no significant change. Despite the absence of a significant increase in PT, the observed increase in V_max_ may reflect optimization for power to some extent, suggesting that GR has a positive effect that enhances the mechanical efficiency of MG. We observed a significant increase in ∆L and ∆θ of the MG in both groups after GR. Specifically, ∆L increased by 30.1% (GR) and 25.4% (CON), and ∆θ increased by 44.9% (GR) and 23.0% (CON). During active muscle contraction, fascicle shortening generates force. Meanwhile, the accompanying fascicle rotation adjusts the direction of muscle force, contributing to the efficient generation of force in various directions. The rotation during fascicle shortening provides a certain degree of freedom, which affects muscle strength and the ability to generate force (Lieber and Friden, 2000). This result suggested that GR leads to changes in ∆L and ∆θ of MG, which may contribute to the rapid generation of sufficient force to support body weight and generate propulsion during the stance phase of running. Considering that muscle behavior also affects the metabolic costs of running (Fletcher et al., 2013), future research could consider combining a contactless monitoring system for monitoring energy expenditure (Huang et al., 2024).

Several limitations remain in this study. Owing to the unique and crucial role of the MG in the plantar flexor muscle group, the study was limited to the MG as the target muscle. Future research may consider extending the focus to other plantar flexor muscles, such as the soleus and lateral gastrocnemius. Only the *in vivo* ultrasound and mechanical characteristics of MG during MVIC were examined, and the muscle behavior and mechanical characteristics during running should be explored in the future. The study recruited only male runners, so the influence of gender on the training effect remains unclear. Furthermore, the duration of GR in this study was 12 weeks, and no follow-up was conducted to assess the maintenance of GR. Therefore, future research could explore gender differences in behavior and mechanical properties of lower limb muscles during running, as well as the long-term effects of periodic training.

## 5 Conclusion

After 12-week gait retraining, significant increases were observed in the rate of torque development within the early 50 ms, as well as in normalized fascicle length and maximal fascicle shortening velocity of the medial gastrocnemius. These findings suggest that gait retraining positively influences the architecture and contraction behavior of the medial gastrocnemius, thereby improving the ability to rapidly develop strength and muscle contraction velocity. Therefore, rearfoot strikers could consider gait retraining as a strategy to potentially improve muscle function and mechanical efficiency, with a particular focus on enhancing the capacity of the medial gastrocnemius to generate and transmit force as well as improving rapid contraction ability. Furthermore, future investigations should delve deeper into understanding the behavior of the medial gastrocnemius during running *in vivo*.

## Data Availability

The raw data supporting the conclusion of this article will be made available by the authors, without undue reservation.
